# Change They Can't Find: Change Blindness in Chimpanzees during a Visual Search Task

**DOI:** 10.1068/i0708sas

**Published:** 2015-04-01

**Authors:** Masaki Tomonaga, Tomoko Imura

**Affiliations:** Primate Research Institute, Kyoto University, Inuyama, Aichi, Japan; Department of Information Culture, Niigata University of International and Information Studies, Niigata, Japan

**Keywords:** change blindness, chimpanzees, visual search

## Abstract

Although considerable advances have been made in the study of change blindness in humans, research regarding change blindness in nonhuman animals has been rare thus far. Indeed, we do not know whether chimpanzees, our closest evolutionary relatives, experience difficulty detecting changes in a stimulus when presentations are separated by blank displays. This study demonstrated that chimpanzees showed severe difficulties in detecting changes in a flicker-type visual search task, and these results are discussed in relation to the adaptive significance of change detection (e.g. the relationship between change blindness and vigilance behaviour).

It is well known that humans often experience severe difficulty finding changes in visual scenes, including when they occur during eye movements, blinks and brief blank displays. This phenomenon, which is known as change blindness (e.g. O'Regan, Deubel, Clark, & Rensink, 2000; Rensink, Clark, & O'Regan, 1997; Simons, 1996; Simons & Rensink, 2005), has captured the attention of many researchers working in the domains of visual perception and memory. Change blindness is also clearly important for nonhuman animals, as this phenomenon seems to constitute a critical contributor to vigilance behaviour (cf. Kutsukake, 2007; Tada, Omori, Hirokawa, Ohira, & Tomonaga, 2013). Despite the adaptive significance of change detection, surprisingly few empirical studies have been conducted regarding change blindness in nonhuman animals, for example, macaques (Cavanaugh & Wurtz, 2004; Landman, Spekreijse, & Lamme, 2004). These studies reported important roles of V1 and subcortical areas in the change detection under the “one-shot” paradigm where the single alternation was presented (Rensink, 2002), but nothing was examined under tasks using more dynamic displays such as a flicker-type visual search task (Rensink, 2000). Furthermore, change blindness is a useful “tool” for studying dynamic scene perception, visual memory and consciousness from a comparative–cognitive perspective. Studies with chimpanzees are quite important to bridge the data between macaques and humans both from neurophysiological and comparative–cognitive perspectives. To this end, we examined change blindness in chimpanzees using a visual search task.

Three adult female chimpanzees participated in the present experiment (Figure 1a). They lived in an environmentally enriched environment with the other 11 chimpanzees and had a long history of participation in visual search tasks (Matsuzawa, 2006; Tomonaga, 2001; Tomonaga & Imura, 2010). Forty-eight line-drawings (40 × 40 mm in size) were used as stimuli, and experimental sessions were conducted in an experimental booth designed for the chimpanzees. Using a 21-in CRT monitor with a touchscreen, the chimpanzees were initially trained to participate in a visual search task in which they were required to touch a target stimulus that changed while they were watching (i.e. a blank screen did not interrupt the change in the stimulus), which was presented among static distractors (no-blank condition, Figure 1b). After reaching the criterion (more than 90% correct; on average, 774 trials were needed to reach the criterion), we introduced two conditions involving white masking displays (Figure 1c, Figure 1d). Under the blank condition, a blank display was inserted between stimulus changes. Under the control-blank condition, a blank display was inserted after the stimulus changed, which allowed participants to view the change process. The latter condition was introduced to examine the possibility that worse performance under the blank condition was due merely to the insertion of a distracting masking stimulus. The number of stimuli in the search display (i.e. search display) varied across three, six or nine. We prepared three types of stimulus changes: presence/absence (the target stimulus was repeatedly turned on and off), positional shift (the position of the target was repeatedly shifted to 10 mm) and identity change (the target identity was repeatedly changed). The duration of the search display (on-time) varied between 90 ms and 320 ms, whereas the duration of the blank display (off-time) varied between 90 ms and 180 ms; thus, four on- and off-time combinations were used. Each session consisted of 108 trials, and each chimpanzee participated in 32 sessions. The care and use of the chimpanzees adhered to the guidelines of the Primate Research Institute, Kyoto University, and the experimental protocol was approved by the Animal Experimentation Committee of Kyoto University.

**Figure 1.**
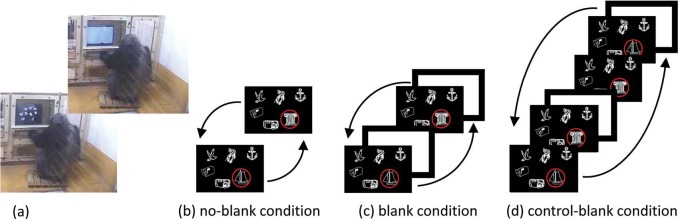
(a) An adult female chimpanzee, Chloe, performing a visual search task. (b–d) Three types of trials.

All chimpanzees produced very similar patterns of results (Figure 2). Four-way analyses of variance (ANOVAs) (blank type x display size x change type x on–off combination) were conducted to analyse data regarding error rates and response times (RTs). Chimpanzees performed significantly more poorly under the blank condition than under the other two control conditions (error rate: *F*(2,4) = 757.8, *p* < 0.001; RT: *F*(2,4) = 53.7, *p* = 0.001), and the effect of display size was significant under only the blank condition (error rate: *F*(2,12) = 108.6, *p* < 0.001; RT: *F*(2,12) = 94.1, *p* < 0.001). The effect of change type was also significant under the blank condition (error rate: *F*(2,12) = 65.8, *p* < 0.001; RT: *F*(2,12) = 15.0, *p* < 0.001). In addition, detection involving positional shift (error rate = 55.8%, RT = 1864 ms) was significantly more difficult (*p* < 0.01) than was detection involving presence/absence (31.6%, 1642 ms) and identity change (45.6%, 1696 ms) conditions under the blank condition. In terms of error rates, under the blank condition, the effects of the on–off combination (*F*(3,18) = 43.3, *p* < 0.001) and of the on-time duration (*p* < 0.001) were significant, but that of the of-time duration was not. With regard to RTs, the main effect of the on–off combination was significant (F(3,6) = 21.3, *p* = 0.001), but only the on-time duration had a significant effect (*p* < 0.01).

**Figure 2.**
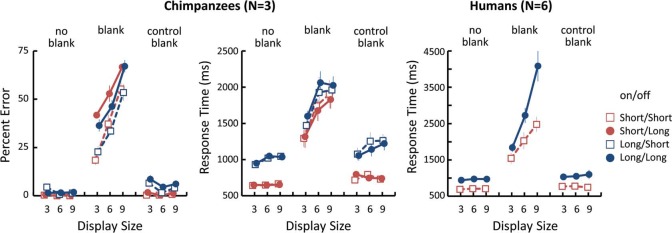
Results of the experiment. Left: error rate in chimpanzees; centre: response times for correct trials in chimpanzees; centre: response times for correct trials in humans. Error bars showed SEMs across participants. All data were averaged across change types.

For comparison, six adult humans participated in the same experiments. Each participant was given two 216-trial sessions in which only the two types of on–off combinations appeared. They exhibited almost no errors (99.4% correct). As shown in the right of Figure 2, humans showed a similar pattern of RTs to chimpanzees. All the main effects were significant (blank type: *F*(2,10) = 82.4, *p* < 0.001; display size: *F*(2,10) = 56.0, *p* < 0.001; change type: *F*(2,10) = 12.7, *p* < 0.01; on–off: *F*(1,5) = 93.7, *p* < 0.001), and four-way interaction was also significant (*F*(8,40) = 3.53, *p* < 0.01). Under the blank condition, as in chimpanzees, detection involving positional shift (3420 ms) was significantly more difficult (*p*'s < 0.001) than was detection involving presence/absence (1627 ms) and identity change (2310 ms) conditions.

Overall, chimpanzees clearly demonstrated considerable difficulty finding changes in the search display of a flicker-type visual search task when the changes were separated by a blank display. Additional tests clearly showed that this result was quite similar to those with humans (cf. Rensink, 2000). Furthermore, when the changes did not occur before and after the blank display, the performances were comparable to those when no blank display was inserted. Thus, mere exposure to the white display could not explain the current results under the blank condition. As in humans, display size exerted an effect on the RTs of chimpanzees. Although the slope was not linear due to the floor effect of accuracy, it suggests that detecting changes demands attention in chimpanzees as well as in humans (Rensink, 2000). We found that positional shifts were the most difficult to detect for the chimpanzees. This can be explained by the “amount” of change. In comparison with the other two types of changes, which involved complete changes in the stimulus, the positional shift condition involved the maintenance of the identity of the stimulus.

On the basis of the current results, future research should examine the various aspects of change blindness in chimpanzees, including the effect of memory capacity, attention, top-down control and so on (Simons & Rensink, 2005). It would also be interesting to examine the relationship between change blindness and vigilance behaviour from a comparative perspective (cf. Kutsukake, 2007; Tada et al., 2013).

CHANGE we can't find, they CAN'T either.
